# Perturbations in the Primary Metabolism of Tomato and *Arabidopsis thaliana* Plants Infected with the Soil-Borne Fungus *Verticillium dahliae*

**DOI:** 10.1371/journal.pone.0138242

**Published:** 2015-09-18

**Authors:** Anja Buhtz, Katja Witzel, Nadine Strehmel, Jörg Ziegler, Steffen Abel, Rita Grosch

**Affiliations:** 1 Leibniz Institute of Vegetable and Ornamental Crops, Theodor-Echtermeyer-Weg 1, 14979 Grossbeeren, Germany; 2 Leibniz Institute of Plant Biochemistry, Weinberg 3, 06120 Halle/Saale, Germany; University of Nebraska-Lincoln, UNITED STATES

## Abstract

The hemibiotrophic soil-borne fungus *Verticillium dahliae* is a major pathogen of a number of economically important crop species. Here, the metabolic response of both tomato and *Arabidopsis thaliana* to *V*. *dahliae* infection was analysed by first using non-targeted GC-MS profiling. The leaf content of both major cell wall components glucuronic acid and xylose was reduced in the presence of the pathogen in tomato but enhanced in *A*. *thaliana*. The leaf content of the two tricarboxylic acid cycle intermediates fumaric acid and succinic acid was increased in the leaf of both species, reflecting a likely higher demand for reducing equivalents required for defence responses. A prominent group of affected compounds was amino acids and based on the targeted analysis in the root, it was shown that the level of 12 and four free amino acids was enhanced by the infection in, respectively, tomato and *A*. *thaliana*, with leucine and histidine being represented in both host species. The leaf content of six free amino acids was reduced in the leaf tissue of diseased *A*. *thaliana* plants, while that of two free amino acids was raised in the tomato plants. This study emphasizes the role of primary plant metabolites in adaptive responses when the fungus has colonized the plant.

## Introduction

Plants are continuously exposed to a range of pathogenic microorganisms, both above and below ground, and have evolved various means to first recognize them and then to defend against infection. Recognition of pathogens is related with massive reprogramming of the plant cell metabolism [1]. The initial phase of the host/pathogen interaction includes the host's perception of pathogen-associated molecular patterns by a specialized group of receptors [2], the erection of lignin- or callose-based structural barriers [3], and the synthesis of reactive oxygen species [4] and phytoalexins [5]. Investment in defence makes the plant ecologically successful, but is thought to reduce plant fitness in an enemy-free environment. Such fitness cost may result from allocation of costs to defence which cannot be used for growth or other physiological processes. Considerable improvement in our understanding of the plant defence response has been made in recent years [6–8], but as yet little is known concerning the underlying metabolic pathways required for growth and regulation of plant defence reactions. The assumption is that the role of the primary metabolism is to provide the energy required for the defence response [9], but this ignores major differences between (hemi)biotrophic and necrotrophic pathogens: the former rely on living host tissue, while the latter first kill the host cells and then obtain their nutrition from dead tissue. A further weakness of the general model is that while much of the living aerial part of the plant is concerned with the accumulation of assimilates, this is not the case for the root. The impact on the host's primary plant metabolism of infection by a leaf pathogen has been more fully characterized than the equivalent impact of pathogens that infect the root system and spread systemically within the below- and above-ground plant parts [10].

Fungi belonging to the genus *Verticillium* are of major commercial importance, since they can attack a broad range of plant species [11–15]. The fungi invade the host via the roots and then spread systemically, causing stunting, chlorosis and wilting; their overall impact is detrimental to both economic yield and end use quality [16,17]. The pathogen locates the root by sensing specific root exudates, and once established within the root, invades the xylem in the form of conidia [18–21]. Polysaccharide lyases are secreted by the pathogen to overcome the plant defence structures and enable proliferation in the xylem [22,23], resulting in stunting, chlorosis and wilting of host plants at progressed disease development [24,25]. The host's transcriptional [26–33] and proteomic [34–38] responses to infection have been well covered in the literature.

The current study focuses on the effect on the host's (tomato and *Arabidopsis thaliana*) primary metabolism to infection by *Verticillium dahliae*. Systemic plant responses, including a reprogrammed carbohydrate and nitrogen metabolism, as well as depressed photosynthesis- and respiration-related processes reflect the plasticity in adapting to pathogenic colonisation. Hence, we expect a measurable influence of pathogen infection on the primary plant metabolism. Non-targeted metabolite profiling and targeted analyses are utilized in our study to understand the complexity of physiological changes such as assimilate partitioning or source-sink regulation, which might be the result of energy-consuming defence responses or the niche establishment of the pathogen. We have selected genotypes of two plant species from Solanaceae (*Solanum lycopersicum* cv. Hildares) and Brassicaceae (*Arabidopsis thaliana* accession Ler-0) in order to gain insight into conserved or divergent metabolic responses towards *Verticillium* infestation. Metabolites were profiled by a combination of gas chromatography coupled to mass spectrometry (GC-MS) and liquid chromatography coupled to electrospray ionisation tandem mass spectrometry (LC—ESI—MS/MS) using multiple reaction monitoring (MRM). The analysis has identified a number of both host species-specific and shared effects on the primary metabolism.

## Materials and Methods

### Plant material and growth conditions

Surface-sterilized tomato cv. Hildares seed (Hild Samen GmbH, Marbach, Germany) was germinated on moist sand for 11–12 days under greenhouse conditions (24±3°C day / 20°C night) and transplanted into pots (9 × 9 × 10 cm) containing a 1:1 mixture of sand and grit (Euroquarz GmbH, Laußnitz, Germany), which were moved into a controlled environment chamber set to give a 16 h photoperiod (400 μmol s^-1^ m^-2^), a day/night temperature regime of 25°C/20°C and a relative humidity of 72% during the day and 80% during the night. The pots were watered on a daily basis with B’cuzz Hydro A+B nutrient solution (ATAMI, Rosmalen, Netherlands). *A*. *thaliana* accession Ler-0 seed (kindly provided by L. Westphal, Leibniz Institute of Plant Biochemistry, Halle, Germany) was germinated in soil and then transplanted after two weeks into sand. The plants were watered regularly with a nutrient solution [39] and were exposed to an 8 h photoperiod (300 μmol m^-2^ s^-1^) at 20/18°C.

### Fungal cultivation and plant inoculation

*V*. *dahliae* isolate GU060637 (kindly provided by V. Grimault, Variety and Seed Study and Control Group GEVES, Angers, France) was maintained on potato dextrose agar (PDA, Merck, Darmstadt, Germany) in petri dishes. Mycelial suspensions were prepared as described by Witzel et al. [40], with minor modifications. Briefly, each flask filled with 100 mL sucrose-sodium nitrate (SSN) media was inoculated with six agar discs (Ø 5 mm) and incubated for one week. Further 200 mL SSN media were added with subsequent incubation for two additional weeks. Afterwards mycelium was filtrated, blended and centrifuged (2 min, 13 000 x *g*). The pellet was twice by resuspension in sterile tap water. Conidia were counted in a Thoma chamber to allow the inoculum concentration to be adjusted to 10^7^ (tomato) or 10^6^ (*A*. *thaliana*) per mL. Tomato (1–2 leaf stage) and *A*. *thaliana* (two week old) seedlings were watered either with 20 mL (tomato) and 10 mL (*A*. *thaliana*) of conidial suspension. As a control, a set of pots was treated in the same way, but mock-inoculated with sterile tap water. Following the treatment, the plants were allowed to grow for up to a further three weeks. Each experiment was replicated twice and the pots were arranged as a randomized complete block.

### Plant tissue sampling

The roots of five mock-inoculated and five inoculated tomato plants were harvested seven, 14 and 21 days post inoculation (dpi), and used to perform a quantitative PCR-based assay directed at the pathogen. For metabolite extraction, root and leaf samples (seventh and eighth leaf as a bulk) were harvested at 21 dpi. The roots were washed to remove the sand and then blotted dry with paper towels. Different portions of each root were harvested and combined for a representable sample. Leaf and root materials were snap-frozen in liquid nitrogen, then milled to a fine powder and stored at −80°C until required. For the *A*. *thaliana*-based experiment, the rosette leaves and the roots were harvested 21 dpi in the same way as above. In both experiments, each of the five replicated samples was a bulk of six plants.

### DNA isolation and quantitative real-time PCR (qPCR)

Genomic DNA was isolated from the powdered tomato and *A*. *thaliana* leaf and root tissue following the Doyle [41] protocol, as modified by Tinker et al. [42]. DNA pellets were dissolved in 200 μl nuclease-free water and purified by RNase A digestion adding 15 μl of a 50 μg mL^-1^ RNase A stock solution and additional phenol/chloroform/isoamylalcohol extraction followed by an acidic ethanol precipitation [43]. The resulting DNA was dissolved in 50 μL TE buffer and the concentration of each sample was estimated using a Nano Drop ND-1000 spectrophotometer (Peqlab, Erlangen, Germany), then used as a PCR template. The PCR primer pair targeted at *V*. *dahliae* was VDS [44] (forward primer 5’-CACATTCAGTTCAGGAGACGGA-3’, reverse primer 5’-CCGAAATACTCCAGTAGAAGG-3’) and those targeting the host were ACT (encoding actin, forward primer 5’-GAAATAGCATAAGATGGCAGACG-3’, reverse primer 5’-ATACCCACCATCACACCAGTAT-3’) and TUB (β-tubulin, forward primer 5’-AACCTCCATTCAGGAGATGTTT-3’, reverse primer 5’- TCTGCTGTAGCATCCTGGTATT-3’), as recommended by Løvdal and Lillo [45]. Stability of the selected reference genes was successfully confirmed by calculating reliable M- and CV-value as parameters using the CFX Manager software (Bio-Rad laboratories GmbH, Munich, Germany) according to Vandesompele et al. [46]. Each 15 μL PCR was composed of 7.5 μL 2x SsoAdvanced™ SYBR® Green Supermix (Bio-Rad laboratories GmbH), 4.7 μL nuclease-free water, 0.9 μL of either VDS (10 μM) or TUB or ACT (5 μM) and 1 μL gDNA (50 ng/μL for root samples and 100 ng/μL for leaf DNA). The reactions were processed in an iCycler system device (Bio-Rad laboratories GmbH), in which the initial denaturation step was 95°C/3 min, the amplification step (40 cycles) was 95°C/30 s, 66°C/30 s (VDS) or 61.4°C/30 s (ACT and TUB), and the final extension step was 72°C/30 s. The reactions were denatured by a 10 s exposure to 95°C and then subjected to a melt curve analysis (60–95°C). The cycle threshold number (Ct) measured from three technical replicates was used to derive a mean Ct. Relative abundance of fungal DNA was calculated by normalizing the VDS signal against the ACT and TUB signals, using CFX Manager software (Bio-Rad laboratories GmbH). The estimated quantity of *V*. *dahliae* DNA present was averaged across the two replicated experiments, and so was based on a sample of ten plants. *A*. *thaliana* root and leaf samples were processed for DNA extraction as above and the quantification of *V*. *dahliae* DNA was performed as described by Witzel et al. [40].

### GC-MS analysis of metabolites

Metabolites were extracted from powdered plant material and derivatized prior to GC-MS analysis, employing a protocol based on those described by Roessner et al. [47] and Schauer et al. [48]. In detail, 470 μL 100% methanol plus 20 μL 0.2 mg mL^-1^ ribitol (Sigma-Aldrich, Deisenhofen, Germany) were added to the powdered material (tomato leaf: 50 mg, *A*. *thaliana* leaf: 40 mg) and agitated at 70°C for 15 min. Root extracts were based on, respectively, 120 mg and 40 mg of ground tissue and 10 μL of the ribitol solution. The samples were centrifuged, after which 250 μL chloroform was added to the supernatant. After a 5 min incubation at 37°C, 500 μL pure H_2_O was added, and the mixture was well mixed and then re-centrifuged. An aliquot of the resulting supernatant (tomato leaf: 100 μL, root: 300 μL; *A*. *thaliana* leaf: 50 μL, root: 100 μL) was dried *in vacuo*, and the residue dissolved in 40 μL of 20 mg mL^-1^ methoxyamine hydrochloride in pyridine (Sigma-Aldrich) and left to derivatize for 1.5 h at 30°C. To achieve trimethylsilylation, 70 μL N-methyl-N-[trimethylsilyl] trifluoroacetamide (Macherey-Nagel GmbH & Co. KG, Düren, Germany) plus a 10 μL retention time standard mixture containing nine alkanes (Sigma-Aldrich) was added, and the mixture incubated with agitation at 37°C for 30 min. The constitution of the alkane mixture, which was dissolved in pyridine, was: 0.044% (v/v) n-decane, 0.022% (v/v) n-dodecane / n-pentadecane, 0.022% (w/v) n-octadecane / n-nonadecane / n-docosane / n-octacosane / n-dotriacontane and 0.044% (w/v) n-hexatriacontane.) Aliquots (80 μL) were transferred into an 8 mm GC chromacol glass vial (ABIMED Analysen-Technik GmbH, Langenfeld, Germany). The derivatized extracts were analysed using a 7890A GC system device coupled to a 5975C Inert XL MSD mass selective detector (Agilent Technologies, Santa Clara, USA) by injecting a 1 μL aliquot of the analyte onto a VF-5ms 30x0.25 + 10 m EZ-guard column (Agilent Technologies). The following temperature programme applied: 70°C/5 min, increasing by 9°C per min up to 350°C, 350°C/5 min, decreasing by 10°C per min to 330°C, then 330°C/5 min. Chromatograms were baseline-corrected using MetAlign software [49] and mass spectral data of the two replicated experiments of each plant species were combined according to tissue type for further data processing using TagFinder software [50], which identifies metabolites via a retention time index and a comparison of mass spectra in reference to the Golm Metabolic Database (GMD, gmd.mpimp-golm.mpg.de/).

### Metabolite data statistics and visualization

To determine relative metabolite levels, signal intensities were normalized to the level of the internal standard (ribitol) and were adjusted according to the fresh weight of the plant material used for extraction. To compare results between control and infected plants, normalized signal responses were subjected to a t-test (p ≤ 0.05) implemented in Microsoft Excel 2010 (Microsoft Corporation, Seattle, USA). The concentration of metabolites differentially present in the root and leaf were log2 transformed fold changes calculated by the ratio of the mean metabolite levels of infected and control plants (n = 10) from both experiments and summarized by their relative metabolite levels, log2 ratios and p values.

### Free amino acid profiling

The concentration of free amino acids was obtained by derivatisation with Fmoc-Cl, followed by LC—ESI—MS/MS analysis, as described by Ziegler and Abel [51]. The analysis was based on five replicated 10 mg powdered leaf/root samples per biological replicate. Analysis for statistical significance was done using Student’s t-test implemented in SigmaPlot 12.3 software (SPSS Inc., Chicago, USA).

## Results

### Colonization of tomato and *A*. *thaliana* by *V*. *dahliae*

**Fig 1:**
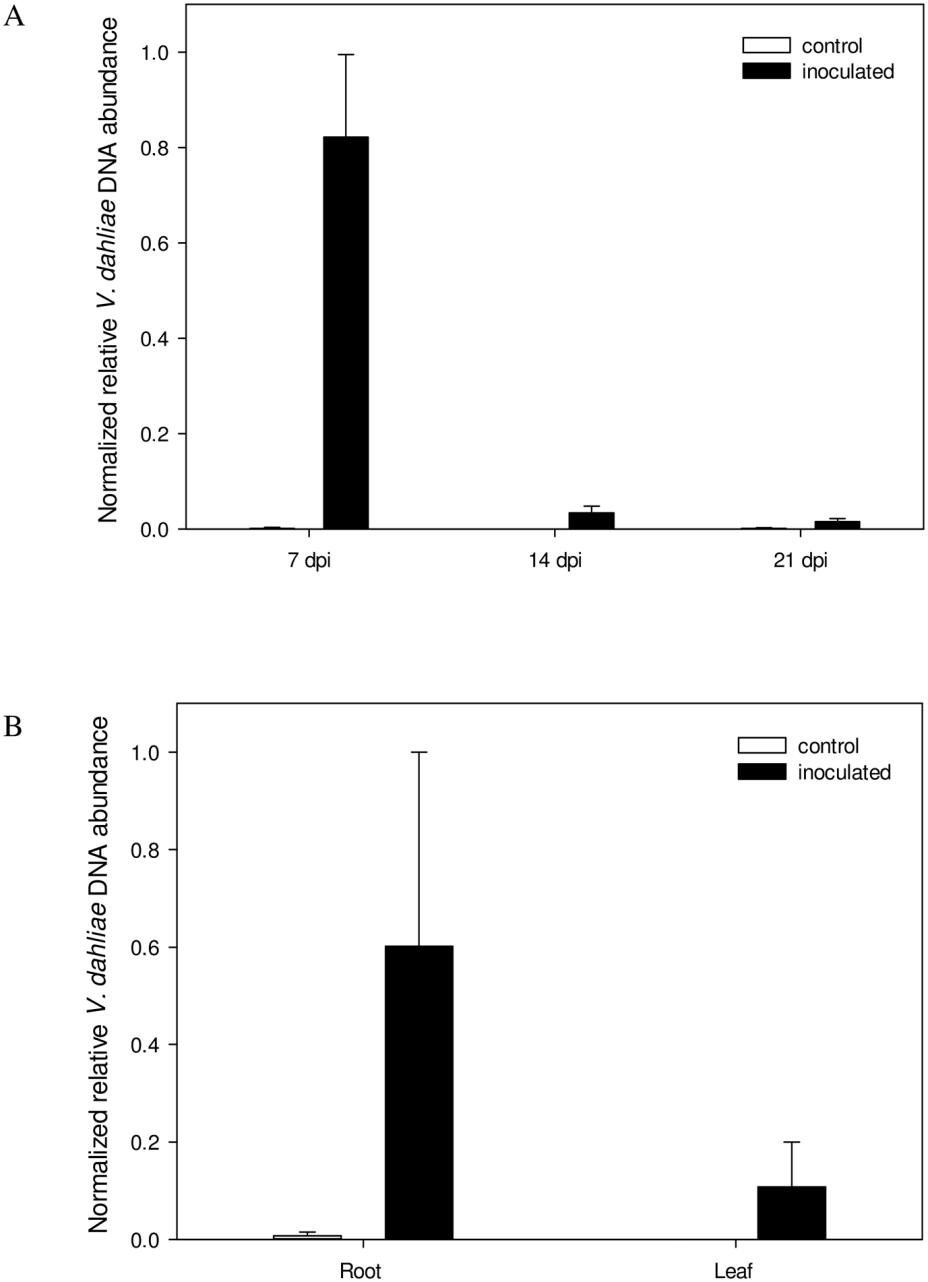
qPCR-based quantification of *V*. *dahliae* in inoculated tomato and *A*. *thaliana* plants. (A) Presence of the pathogen in tomato roots sampled at 7, 14 and 21 dpi. No fungal DNA was detectable in the leaf. (B) Presence of the pathogen in the leaf and root of *A*. *thaliana* sampled at 21 dpi. Values represent means of two independent experiments ± SE, each consisting of 5 and 25 plants for tomato and *A*. *thaliana*, respectively.

The qPCR assay demonstrated that *V*. *dahliae* was present in the tomato root at both 7 and 14 dpi, but was barely detectable by 21 dpi (Fig 1A). No pathogen DNA was recorded in the tomato leaf at any of the time points. The *A*. *thaliana* plants were only sampled at 21 dpi, at which time *V*. *dahliae* DNA was present in both the leaf and root of every plant (Fig 1B), although the quantity of fungal DNA was considerably greater in the root than in the leaf.

### *V*. *dahliae* infection differentially affected the primary metabolism of the two hosts

The untargeted GC-MS analysis identified 78 annotated compounds in the leaf of tomato and 74 in the root. Of the leaf compounds, 14 were quantitatively altered by the infection in plants sampled at 21 dpi, and similarly so were 11 of the root compounds (Table 1). Except for four of the leaf compounds, the identifications were based on matching the mass spectrum and the retention index (reverse match > 500, retention index deviation < 0.5%) using the mass spectral library of the GMD. Eight of the compounds preferentially accumulated in the roots of infected plants were amino acids (aspartic acid, glutamic acid, isoleucine, phenylalanine, proline, serine, threonine and valine) and two were sugar phosphates (glucose-6- phosphate and glycerol-3-phosphate); the abundance of glucose was reduced. In the leaf, the preferentially accumulated compounds also included amino acids (glutamine, isoleucine, threonine, tyrosine, valine) and fumaric acid, while the presence of fructose, glucose, xylose and glucuronic acid was reduced. Four annotated compounds registered as unknown substances in the GMD and therefore without any identity so far were also reduced by the treatment. The equivalent analysis of *A*. *thaliana* identified 55 compounds in the leaf and 42 in the root. The level of seven leaf compounds was significantly enhanced by the presence of *V*. *dahliae*, but none were identified in the root (Table 2). The preferentially accumulated leaf compounds were aspartic acid, xylose, glucuronic acid, ribonic acid, succinic acid, trans sinapic acid and 2-*O*-glycerolglucopyranoside.

**Table 1:** Differentially accumulated metabolites in the tomato leaf and root of plants sampled at 21 dpi, as identified by non-targeted GC-MS profiling. For each metabolite, the content in the non-inoculated and inoculated plants was compared using Student’s t-test (p < 0.05). The extent of the difference in concentration is indicated by log2 of the fold change in metabolite content between inoculated and non-inoculated plants, with the associated p value. The number of trimethylsilyl and methoximated groups is given in parentheses.

Metabolite group	Annotated metabolite	Log2 fold change (inoculated/control)	p value
**Leaf**			
Amino acids	Glutamine [-H_2_O] (2TMS BP)	1.06	0.000
	Isoleucine (2TMS)	0.36	0.023
	Threonine (3TMS)	0.49	0.022
	Tyrosine (3TMS)	0.34	0.038
	Valine (2TMS)	0.47	0.011
Sugars	Fructose (1MeOX, 5TMS BP)	-0.53	0.018
	Glucose (1MeOX, 5TMS BP)	-0.60	0.042
	Glucose (1MeOX, 5TMS MP)	-0.47	0.025
	Xylose (1MeOX, 4TMS MP)	-0.34	0.039
Organic acids	Glucuronic acid (1MeOX, 5TMS MP)	-0.85	0.053
TCA cycle	Fumaric acid (2TMS)	0.39	0.014
Unknown	A181004 NA	-0.70	0.025
	A203003 NA	-0.82	0.052
	A213001 NA	-0.53	0.036
	A268003 NA	-0.32	0.033
**Root**			
Amino acids	Aspartic acid (2TMS)	0.62	0.039
	Glutamic acid (2TMS)	0.44	0.019
	Isoleucine (2TMS)	0.55	0.015
	Phenylalanine (1TMS)	0.40	0.044
	Proline (2TMS)	0.85	0.021
	Serine (3TMS)	1.03	0.009
	Threonine (3TMS)	0.90	0.013
	Valine (2TMS)	0.75	0.009
Sugars	Glucose (1MeOX, 5TMS BP)	-0.29	0.033
Phosphates	Glucose-6-phosphate (6TMS)	0.30	0.025
	Glycerol-3-phosphate (4TMS)	0.24	0.048

**Table 2:** Differentially accumulated metabolites in *A*. *thaliana* leaves harvested from plants at 21 dpi, as identified by GC-MS profiling. For each metabolite, the content in the non-inoculated and inoculated plants was compared using Student’s t-test (p < 0.05). The extent of the difference in concentration is indicated by log2 of the fold change in metabolite content between inoculated and non-inoculated plants, with the associated p value. The number of trimethylsilyl and methoximated groups is given in parentheses.

Metabolite group	Annotated analyte	Log2 fold change (inoculated/control)	p value
Amino acids	Aspartic acid (4TMS)	1.8	0.04
Sugars	Xylose (1MeOX, 4TMS)	1.6	0.05
Organic acids	Glucuronic acid (5TMS)	1.4	0.03
	Ribonic acid (5TMS)	1.2	0.03
TCA cycle	Succinic acid (2TMS)	1.3	0.05
Phenylpropanoid	trans Sinapic acid (2TMS)	1.4	0.04
Glycerol glucoside	2-*O*-Glycerolglucopyranoside (6TMS)	2.3	0.01

### Targeted amino acid analysis

Results of untargeted GC-MS analysis indicated significant alterations in quantity of specific amino acids due to the *V*. *dahliae* inoculation. Therefore, the validation of these results by targeted amino acid analysis was pursued. The targeted amino acid profiling identified 12 compounds in the tomato root which responded positively to infection (Table 3). The analysis confirmed that the levels of aspartic acid, isoleucine, phenylalanine, proline, serine and threonine were all enhanced in infected tissue, but in addition that the levels of arginine, histidine, leucine, lysine and tyrosine were also raised. However, the profiling failed to verify that either glutamic acid or valine were responsive. In the leaf, while the enhancement in the presence of tyrosine was confirmed, it was evident that the concentration of serine also responded to infection. The analysis did not validate the GC-MS based conclusion that the concentrations of glutamine, isoleucine, threonine and valine were enhanced by the presence of the pathogen. The analogous analysis in *A*. *thaliana* identified six responsive amino acids in the leaf and four in the root (Table 4). In the leaf, the concentrations of glutamic acid, histidine, isoleucine, lysine, tryptophan and valine were all reduced by the presence of *V*. *dahliae*, while in the root, the levels of glutamine, histidine, leucine and valine were increased.

**Table 3:** The concentration of individual free amino acids [μmol per g fresh weight] in tomato plants at 21 days post inoculation with *Verticillium dahliae* (+Vd). Values presented as the mean ± SD (n = 2), each replicate consisting of five plants, and analysed as two technical replicates. Asterisks indicate statistically significant differences (p < 0.05) between non-inoculated and inoculated plants.

	Root		Leaf	
	Control	+Vd	Control	+Vd
A—Alanine	0.19±0.17	0.21±0.07	0.66±0.31	0.56±0.22
C—Cysteine	0.08±0.05	0.13±0.10	0.62±0.20	0.56±0.25
D—Aspartic acid	0.47±0.14	0.74±0.28*	1.89±0.43	2.10±0.63
E—Glutamic acid	1.95±0.88	2.36±0.85	2.70±0.65	2.68±0.72
F—Phenylalanine	0.04±0.02	0.06±0.03*	0.11±0.04	0.11±0.04
G—Glycine	0.05±0.04	0.06±0.02	0.13±0.05	0.13±0.06
H—Histidine	0.13±0.04	0.20±0.09*	0.07±0.02	0.09±0.03
I—Isoleucine	0.03±0.01	0.06±0.05*	0.03±0.01	0.04±0.02
K—Lysine	0.06±0.03	0.10±0.05*	0.07±0.02	0.08±0.03
L—Leucine	0.04±0.01	0.10±0.06*	0.03±0.02	0.04±0.01
M—Methionine	0.02±0.00	0.03±0.01*	0.06±0.01	0.06±0.02
N—Asparagine	0.73±0.36	0.69±0.29	0.22±0.08	0.24±0.09
P—Proline	0.18±0.08	0.37±0.24*	0.38±0.25	0.49±0.27
Q—Glutamine	1.09±0.47	1.20±0.41	1.97±0.69	2.47±0.89
R—Arginine	0.03±0.02	0.05±0.02*	0.07±0.02	0.07±0.03
S—Serine	0.17±0.05	0.27±0.10*	0.58±0.17	0.78±0.22*
T—Threonine	0.07±0.02	0.15±0.09*	0.67±0.17	0.60±0.23
V—Valine	0.05±0.03	0.10±0.06	0.07±0.03	0.09±0.04
W—Tryptophan	0.01±0.00	0.02±0.01	0.02±0.01	0.03±0.01
Y—Tyrosine	0.03±0.01	0.05±0.02*	0.03±0.01	0.05±0.02*

**Table 4:** The concentration of individual free amino acids [μmol per g fresh weight] in *A*. *thaliana* plants at 21 days post inoculation with *Verticillium dahliae* (+Vd). Values presented as the mean ± SD (n = 2), each experiment consisting of 30 plants, split into five pools of six plants, and analysed as two technical replicates. Asterisks indicate statistically significant differences (p < 0.05) between non-inoculated and inoculated plants.

	Root		Leaf	
	Control	+Vd	Control	+Vd
A—Alanine	5.40±3.83	6.22±3.13	0.64±0.18	0.55±0.16
C—Cysteine	0.60±0.20	0.74±0.22	0.38±0.19	0.38±0.19
D—Aspartic acid	1.29±0.17	1.50±0.62	3.39±0.74	3.02±0.80
E—Glutamic acid	3.40±0.96	3.58±0.80	5.21±0.87	4.23±0.97*
F—Phenylalanine	0.01±0.00	0.01±0.00	0.01±0.00	0.01±0.00
G—Glycine	0.48±0.16	0.57±0.11	0.23±0.19	0.21±0.23
H—Histidine	0.55±0.10	0.67±0.11*	0.18±0.04	0.14±0.03*
I—Isoleucine	0.05±0.02	0.06±0.01	0.04±0.02	0.03±0.01*
K—Lysine	0.96±0.71	0.87±0.56	0.16±0.07	0.11±0.03*
L—Leucine	0.10±0.03	0.13±0.03*	0.04±0.01	0.03±0.01
M—Methionine	0.09±0.03	0.09±0.02	0.12±0.04	0.10±0.04
N—Asparagine	1.70±0.44	1.95±0.39	1.39±0.29	1.17±0.41
P—Proline	0.15±0.03	0.13±0.04	0.37±0.09	0.30±0.18
Q—Glutamine	4.56±1.89	6.45±1.74*	6.57±1.56	5.73±2.34
R—Arginine	0.54±0.39	0.63±0.33	0.16±0.07	0.13±0.05
S—Serine	1.13±0.49	1.27±0.35	1.34±0.68	1.07±0.61
T—Threonine	0.80±0.27	0.86±0.14	0.55±0.25	0.47±0.22
V—Valine	0.21±0.06	0.27±0.05*	0.12±0.04	0.09±0.02*
W—Tryptophan	0.02±0.01	0.02±0.01	0.02±0.01	0.01±0.00*
Y—Tyrosine	0.12±0.06	0.12±0.03	0.08±0.03	0.07±0.02

The two host species displayed a distinct metabolic reaction to the presence of the pathogen, as reflected by the relatively small number of responsive metabolites in common between them. Intermediates of the tricarboxylic acid (TCA) cycle were accumulated in the leaves of infected plants of both host species. The amino acids histidine and leucine were preferentially accumulated in the root of both hosts. Among the most notable of the differential responses, the content in the leaf of glucuronic acid and xylose was raised in *A*. *thaliana*, but reduced in tomato; and there was zero overlap with respect to the responsive amino acids in the leaf.

## Discussion

*V*. *dahliae* has a broad host range, including species belonging to both the Solanaceae and the Brassicaceae families; for this reason, the present study focused on a host from each of these two commercially important families. The divergent and conserved metabolic reactions on primary metabolism on the two host plants *Arabidopsis* and tomato were compared at stages where the fungus has established itself within the plant without visible disease symptoms. Plants have evolved a variety of ways to deal with pathogen infection; some of these involve a significant investment in energy, while others are more passive [1]. The former inevitably divert energy away from primary metabolic processes, so the expectation was that tissue supporting the growth of *V*. *dahliae* would exhibit perturbations in some of the metabolites associated with primary metabolism caused by the induction of plant defence mechanisms and movement of nutrients from host to pathogen. The induction of various pathogen-related (PR) genes in Arabidopsis was shown already in the early stage of Verticillium infection and depends on ethylene and jasmonic acid-associated signals [52,53]. Own observation (data not shown) and results of Robb et al. [32] demonstrated that tomato root and shoot also upregulated defence-related genes in response to Verticillium colonization. Hence, carbohydrate metabolism plays a vital role in regulating defence responses or inducing pathogen related (*PR*) genes by sugars [54,55]. Indeed we found an accumulation of glucose-6-phosphate (Glc-6-P) in tomato roots, It’s converting enzyme Glc-6-P dehydrogenase (Glc-6-P DH) catalyses a rate-limiting step in the pentose phosphate pathway and the activity seems to be crucial for the proper localization of the key factor NPR1 in salicylic acid defence pathway [56]. Additionally, we observed a higher level of glycerol-3-phosphate (G-3-P) in infected tomato roots. G-3-P levels contribute to basal resistance against *Colletotrichum higginsianum* in *A*. *thaliana* [57,58] and our results indicate that G-3-P could be involved in systemic acquired resistance against Verticillium as well. Also, TCA cycle intermediates were accumulated in the infected leaves of both hosts (fumaric acid in tomato and succinic acid in *A*. *thaliana*), indicating an enhanced demand for energy, supplied by inducing an increase in respiratory activity [1,59]. Intriguingly, for certain of the induced metabolites, a diametrically opposite response was exhibited by the two hosts. Thus, the leaf content of both glucuronic acid and xylose responded negatively in tomato but positively in *A*. *thaliana*. Xylose is a basic building block of the plant cell wall; its precursor glucuronic acid is synthesized from sucrose [60]. Part of the host's defence reaction to fungal colonization of the vascular system is to contain conidia within the xylem via the deposition of suberin on the vascular cell wall, while at the same time in order to maintain a functional xylem, the plant generates new vessel elements [25,61,62]. The accumulation of xylose in infected leaves reflects an increased demand for cell wall components. In tomato, the falls in xylose and glucuronic acid level would be expected to have facilitated the spread of the fungus, while their accumulation in the *A*. *thaliana* leaf would be expected to have restricted it. One of the responses of *A*. *thaliana* to infection by *V*. *longisporum* is an increase in cell wall biomass [35] and it seems that the exclusion of conidia from leaf tissue by erecting a physical barrier is an important defence mechanism to isolate the pathogen.

In both host species, a variety of free amino acids was accumulated in the roots of inoculated plants, in accordance with observations made in a number of plant/pathogen interactions. Among many examples is the interaction of *A*. *thaliana* with its bacterial pathogen *Rhodococcus fascians* [63], where numerous amino acids and other primary compounds were found increased and concomitant transcriptome data indicated a reprogramming of the host primary metabolism to create a sink environment for establishing a bacterial population. Another study relates to sunflower cultivars lacking resistance to *Sclerotinia sclerotiorum*, where infected tissue has been shown to accumulate valine, tyrosine and asparagine, while the equivalent tissues in resistant cultivars do not [64]. Similarly, disease establishment of *Magnaporthe grisea* in *Brachypodium distachyon* was correlated with carbon and nitrogen source sequestration in infected leaves [65]. Metabolic alterations in the host involving free amino acids have been described in the interaction between *A*. *thaliana* and the nematode *Heterodera schachtii* [66]. The accumulation of free amino acids in pathogen-infected roots may reflect an increased demand for carbon. Amino acids can be shuttled into energy-generating pathways such as the TCA cycle, and nitrogen deficiency triggers disease development (reviewed by [1,59]). Then again, higher levels of amino acids are accessed by *V*. *dahliae* as nitrogen source. Both leucine and histidine were preferentially accumulated in the *V*. *dahliae* infected roots of both tomato and *A*. *thaliana*, and *in vitro* growth experiments have shown that these two amino acids are the ones most effectively used by the fungus [67]. Given that free amino acids provide the pathogen with a readily accessible source of nitrogen, a strategy through which the pathogen induces the host to generate a local supply of these compounds will favour its proliferation within the host.

The metabolic perturbations induced by the infection in tomato were more marked than those induced in *A*. *thaliana*, a consequence perhaps of the former's longer life cycle which may force the host to mount a more concerted defence response [68]. The group of amino acids was affected by the colonization in both host plants. It is not known whether the accumulation of amino acids in the infected root results from the ability of the pathogen to reprogramme the host's metabolism, or whether it reflects the host's requirement to generate TCA intermediates from free amino acids. Establishing which of these routes is involved will need further experimentation. Since mounting the full defence response requires a substantial flux of carbon from primary to secondary plant metabolites, a detailed time course of the metabolic response of both disease resistant and susceptible host plants should help to identify marker signatures in the plant-pathogen interaction.
